# International Cost-Effectiveness Analysis of Durvalumab in Stage III Non–Small Cell Lung Cancer

**DOI:** 10.1001/jamanetworkopen.2024.13938

**Published:** 2024-05-30

**Authors:** Samuel A. Kareff, Sunwoo Han, Benjamin Haaland, Chinmay J. Jani, Rhea Kohli, Pedro Nazareth Aguiar, Yiqing Huang, Ross A. Soo, Ángel Rodríguez-Perez, Jesús García-Foncillas, Manuel Dómine, Gilberto de Lima Lopes

**Affiliations:** 1 University of Miami Sylvester Comprehensive Cancer Center/Jackson Memorial Hospital, Miami, Florida; 2 University of Miami Miller School of Medicine, Miami, Florida; 3 Pentara Corporation, Salt Lake City, Utah; 4 Case Western University School of Medicine, Cleveland, Ohio; 5 Oncoclínicas, São Paulo, São Paulo, Brazil; 6 National University Cancer Institute, Singapore; 7 Fundación Jiménez Díaz University Hospital, Madrid, Spain

## Abstract

**Importance:**

Standard of care for unresectable locally advanced non–small cell lung cancer (NSCLC) involves definitive chemoradiotherapy followed by maintenance therapy with durvalumab. However, the cost of durvalumab has been cited as a barrier to its use in various health systems.

**Objective:**

To evaluate the cost-effectiveness of durvalumab vs placebo as maintenance therapy in patients with unresectable stage III NSCLC from 4 international payer perspectives (US, Brazil, Singapore, and Spain).

**Design, Setting, and Participants:**

In this economic evaluation, a Markov model was designed to compare the lifetime cost-effectiveness of maintenance durvalumab for unresectable stage III NSCLC with that of placebo, using 5-year outcomes data from the PACIFIC randomized placebo-controlled trial. Individual patient data were extracted from the PACIFIC, KEYNOTE-189, ADAURA, ALEX, and REVEL randomized clinical trials to develop a decision-analytic model to determine the cost-effectiveness of durvalumab compared with placebo maintenance therapy over a 10-year time horizon. Direct costs, adverse events, and patient characteristics were based on country-specific payer perspectives and demographic characteristics. The study was conducted from June 1, 2022, through December 27, 2023.

**Main Outcomes and Measures:**

Life-years, quality-adjusted life years (QALYs), lifetime costs, and incremental cost-effectiveness ratios (ICERs) were estimated at country-specific willingness-to-pay thresholds ([data reported in US$] US: $150 000 per QALY; Brazil: $22 251 per QALY; Singapore: $55 288 per QALY, and Spain: $107 069 per QALY). One-way and probabilistic sensitivity analyses were performed to account for parameters of uncertainty. A cost-threshold analysis was also performed.

**Results:**

The US base-case model found that treatment with durvalumab was associated with an increased cost of $114 394 and improved effectiveness of 0.50 QALYs compared with placebo, leading to an ICER of $228 788 per QALY. Incremental cost-effectiveness ratios, according to base-case models, were $141 146 for Brazil, $153 461 for Singapore, and $125 193 for Spain. Durvalumab price adjustments to the PACIFIC data improved cost-effectiveness in Singapore, with an ICER of $45 164. The model was most sensitive to the utility of durvalumab.

**Conclusions and Relevance:**

In this cost-effectiveness analysis of durvalumab as maintenance therapy for unresectable stage III NSCLC, the therapy was found to be cost-prohibitive from the perspective of various international payers according to country-specific willingness-to-pay thresholds per QALY. The findings of the study suggest that discounted durvalumab acquisition costs, as possible in Singapore, might improve cost-effectiveness globally.

## Key Points

**Question:**

Is maintenance durvalumab therapy cost-effective from the perspective of multiple payers for the treatment of unresectable stage III non–small cell lung cancer?

**Findings:**

In this economic evaluation Markov model simulation, durvalumab was not cost-effective from the perspective of the US, Brazil, Singapore, or Spain at current pricing compared with established health economics thresholds. Discounted acquisition costs in Singapore facilitated cost-effectiveness.

**Meaning:**

The findings of this study suggest that value-based pricing is an alternative model that industry might adapt to inform global pricing strategies that minimize financial toxicity and increase global access to oncology drugs.

## Teaser

This economic evaluation compares the cost-effectiveness of durvalumab for maintenance treatment of stage III non–small cell lung cancer (NSCLC) across the US, Brazil, Singapore, and Spain.

## Introduction

The standard-of-care treatment paradigm for unresectable stage III non–small cell lung cancer (NSCLC) consists of consolidative chemoradiotherapy followed by maintenance durvalumab immunotherapy, as solidified by the PACIFIC trial.^1^ Five-year long-term outcomes further assessing progression-free survival (PFS) and overall survival (OS) confirm the regimen’s superiority, ultimately leading to absolute 9.5% OS and 14.1% PFS improvements.^2^

While the regimen has high-quality, phase 3 trial evidence backing its use, limitations of its global uptake include cost, health system approvals, access, and patient eligibility. For example, the European Medicines Agency only approved the regimen for patients whose tumors harbor programmed-death ligand-1 (PD-L1) scores of 1% or higher.^3^

To our knowledge, cost-effectiveness has not been assessed in a formal, international fashion incorporating the most recent long-term data. Given multiple limitations to the global use of maintenance durvalumab in this setting, we conducted a cost-effectiveness analysis according to 4 country perspectives: (1) the US, (2) Brazil, (3) Singapore, and (4) Spain.

## Methods

### Markov Model Structure

The study was conducted from June 1, 2022, through December 27, 2023. As this analysis does not directly involve human participants, it is exempt from institutional review board or ethics review per the Common Rule. The analysis was performed according to the Consolidated Health Economic Evaluation Reporting Standards (CHEERS) reporting guideline.

We created a Markov decision-analytic model using clinical data from the PACIFIC^1^ randomized clinical trial comprising consolidative chemoradiotherapy, followed by 1 year of maintenance durvalumab immunotherapy, or consolidative chemoradiotherapy only. Patients in both arms received standard-of-care therapy at the first (POD1) and second (POD2) instances of disease progression according to *EGFR*, *ALK*, and PD-L1 tumor proportion score biomarker status on a 10-year horizon (Table 1).^1,2,4,5,6,7,8,9,10,11,12,13,14,15,16^According to current European Medicines Agency approval, patients in the Spanish model received durvalumab only if the tumor proportion score was 1% or greater.^3^

**Table 1.** Markov Model List of Variables, Input, and Corresponding References for US Base Case

Variable	Input	Source
Dose sizes		
Durvalumab	10 mg/kg	NA
Pembrolizumab plus carboplatin plus pemetrexed	200 mg plus AUC plus 500 mg /m^2^	NA
Pembrolizumab plus carboplatin plus paclitaxel	200 mg plus AUC plus 200 mg /m^2^	NA
Carboplatin plus pemetrexed	AUC plus 75 mg/m^2^	NA
Carboplatin plus paclitaxel	AUC plus 200 mg /m^2^	NA
Docetaxel	75 mg/m^2^	NA
Osimertinib	80 mg	NA
Alectinib	600 mg	NA
Drug cycles and sequence		
Durvalumab	Every 2 wk up to 24 cycles	NA
Pembrolizumab plus carboplatin plus pemetrexed (POD1, PDL1 positive and unknown, nonsquamous histologic findings)	Every 3 wk up to 4 cycles total; pembrolizumab and pemetrexed combined up to 31 cycles	NA
Pembrolizumab plus carboplatin plus (nab)-paclitaxel (POD1, PDL1 positive and unknown, squamous histologic findings)	Every 3 wk up to 4 cycles total; pembrolizumab alone up to 31 cycles	NA
Carboplatin plus pemetrexed (POD1, PDL1 negative, nonsquamous histologic findings)	Every 3 wk up to 4 cycles; pemetrexed alone up to 29 cycles	NA
Carboplatin plus (nab)-paclitaxel (POD1, PDL1 negative, squamous histologic findings)	Every 3 wk up to 4 cycles	NA
Docetaxel (PD2)	Every 3 wk up to 6 cycles	NA
Osimertinib (*EGFR* mutant, POD1 only)	Daily up to 36 mo	NA
Alectinib (*ALK* positive, POD1 only)	Twice daily up to 36 mo	NA
Dose timing		
Durvalumab	1 h	NA
Pembrolizumab	0.5 h	NA
Carboplatin	0.5 h	NA
Docetaxel	1 h	NA
Pemetrexed	0.17 h	NA
(Nab)-paclitaxel	0.5 h	NA
Durvalumab adverse events, % of patients experiencing grades 3 or 4 toxic effects on trial		
Fatigue	0.002	Antonia et al,^1^ 2017; Antonia et al,^4^ 2018
Dyspnea	0.015	Antonia et al,^1^ 2017; Antonia et al,^4^ 2018
Pneumonia	0.044	Antonia et al,^1^ 2017; Antonia et al,^4^ 2018
Pneumonitis	0.034	Antonia et al,^1^ 2017; Antonia et al,^4^ 2018
Diarrhea	0.006	Antonia et al,^1^ 2017; Antonia et al,^4^ 2018
Anemia	0.029	Antonia et al,^1^ 2017; Antonia et al^4^ 2018
Placebo adverse events, %		Antonia et al,^1^ 2017; Antonia et al^4^ 2018
Fatigue	0.013	Antonia et al,^1^ 2017; Antonia et al^4^ 2018
Dyspnea	0.026	Antonia et al,^1^ 2017; Antonia et al^4^ 2018
Pneumonia	0.038	Antonia et al,^1^ 2017; Antonia et al^4^ 2018
Pneumonitis	0.021	Antonia et al,^1^ 2017; Antonia et al^4^ 2018
Diarrhea	0.013	Antonia et al,^1^ 2017; Antonia et al^4^ 2018
Anemia	0.034	Antonia et al,^1^ 2017; Antonia et al^4^ 2018
Pembrolizumab plus platinum doublet adverse events		
Fatigue	0.057	Gandhi et al,^5^ 2018
Diarrhea	0.052	Gandhi et al,^5^ 2018
Neutropenia	0.158	Gandhi et al,^5^ 2018
Dyspnea	0.037	Gandhi et al,^5^ 2018
Anemia	0.163	Gandhi et al,^5^ 2018
Docetaxel adverse events		
Fatigue	0.1	Garon et al,^6^ 2014
Diarrhea	0.03	Garon et al,^6^ 2014
Dyspnea	0.08	Garon et al,^6^ 2014
Stomatitis	0.02	Garon et al,^6^ 2014
Vomiting	0.02	Garon et al,^6^ 2014
Osimertinib adverse events		
Diarrhea	0.03	Soria et al,^7^ 2018
Fatigue	0.01	Soria et al,^7^ 2018
Dyspnea	0.01	Soria et al,^7^ 2018
Rash	0.01	Soria et al,^7^ 2018
Anemia	0.03	Soria et al,^7^ 2018
Alectinib adverse events		
Anemia	0.05	Peters et al,^8^2017
Photosensitivity reaction	0.01	Peters et al,^8^2017
Patient characteristics		
Body weight (female), kg	77.5	National Center for Health Statistics,^9^2021
Body weight (male), kg	90.6	National Center for Health Statistics,^9^2021
Body surface area (female, Dubois formula), m^2^	1.82	National Center for Health Statistics,^9^2021
Body surface area (male, Dubois formula), m^2^	2.06	National Center for Health Statistics,^9^2021
Serum creatinine (female), mg/dL	0.96	Jones et al,^10^1998
Serum creatinine (male), mg/dL	1.16	Jones et al,^10^1998
Squamous histologic findings, %	45.7	Antonia et al,^1^ 2017; Antonia et al,^4^ 2018
Nonsquamous histologic findings, %	54.3	Antonia et al,^1^ 2017; Antonia et al,^4^ 2018
Median (range) age, y	64 (23-90)	Antonia et al,^1^ 2017; Antonia et al,^4^ 2018
Female, %	29.9	Antonia et al,^1^ 2017; Antonia et al,^4^ 2018
Male, %	70.1	Antonia et al,^1^ 2017; Antonia et al,^4^ 2018
PD-L1 TPS≥1%, %	42.5	Spigel et al,^2^ 2022
PD-L1 TPS<1%, %	20.8	Spigel et al,^2^ 2022
PD- L1 TPS unknown, %	36.7	Spigel et al,^2^ 2022
*EGFR* mutant, %	6	Antonia et al,^1^ 2017
*ALK* rearranged, %	1.12	Spigel et al,^2^ 2022
Costs (2022 $)		
Durvalumab, 1 mg	7.71	Centers for Medicare and Medicaid Services,^11^ 2022
Pembrolizumab, 1 mg	53.7	Centers for Medicare and Medicaid Services,^11^ 2022
Pemetrexed, 1 mg	7.64	Centers for Medicare and Medicaid Services,^11^ 2022
Carboplatin, 50 mg	2.77	Centers for Medicare and Medicaid Services,^11^ 2022
Docetaxel, 1 mg	0.47	Centers for Medicare and Medicaid Services,^11^ 2022
(Nab)-paclitaxel, 1 mg	14.87	Centers for Medicare and Medicaid Services,^11^ 2022
Osimertinib, 80 mg, monthly	17 079.18	Centers for Medicare and Medicaid Services,^12^ 2022
Alectinib, 600 mg, monthly	18 160.67	Centers for Medicare and Medicaid Services,^12^ 2022
Imaging/surveillance (ie, PET/CT; CPT 78816)	2045.91	Centers for Medicare and Medicaid Services,^13^ 2022
Drug administration/h (ie, chemotherapy infusion 1 h; CPT 96413)	205.43	Centers for Medicare and Medicaid Services,^13^ 2022
Immunohistochemical testing (1st stain; CPT 88342)	141.4	Centers for Medicare and Medicaid Services,^13^ 2022
Radiotherapy (ie, 60 Gy, CPT 77301)	2036.3	Centers for Medicare and Medicaid Services,^13^ 2022
Monthly best supportive care (CPT 99498)	80.91	Centers for Medicare and Medicaid Services,^13^ 2022
Death costs (non–small cell lung cancer)	13 273	Criss et al,^14^ 2019
Death costs (other cause)	10 492	Criss et al,^14^ 2019
Fatigue	16 185	Georgieva et al,^15^ 2018
Dyspnea	6018	Wong et al,^16^ 2018
Pneumonia	9941	Wong et al,^16^ 2018
Diarrhea	3265	Wong et al,^16^ 2018
Anemia	4353	Wong et al,^16^ 2018
Pneumonitis	9941	Wong et al,^16^ 2018
Neutropenia	5321	Wong et al,^16^ 2018
Stomatitis	1695	Wong et al,^16^ 2018
Vomiting	895	Wong et al,^16^ 2018
Rash/photosensitivity reaction	940	Wong et al,^16^ 2018
Utilities		
Progression-free survival	0.79	Criss et al,^14^ 2019
Metastatic/progressive disease (ie, POD1, POD2)	0.76	Criss et al,^14^ 2019
Death	0	Criss et al,^14^ 2019

Abbreviations: AUC, area under the curve; CPT, Current Procedural Terminology (billing codes); CT, computed tomography; NA, not applicable; PDL1, programmed death-ligand 1; PET, positron emission tomography; POD1, first instance of disease progression; POD2, second instance of disease progression; TPS, tumor proportion score.

SI conversion factor: To convert serum creatinine to micromoles per liter, multiply by 88.4.

The model simulated 1000 patients over 1-month (ie, 30-day) cycles. Patients transitioned state-to-state according to survival data from the PACIFIC,^1^ KEYNOTE-189,^5^ FLAURA,^7^ ALEX,^8^ and REVEL^6^ randomized clinical trials. For example, patients progressing on the durvalumab or placebo treatment arms of PACIFIC would represent the first instance of disease progression (POD1), followed by a transition to the treatment arm of the KEYNOTE-189, FLAURA, or ALEX paradigms. In rare instances a patient might transition directly from the PACIFIC data directly to the second instance of disease progression (POD2) of the treatment arm of the REVEL paradigm, in line with both previous Markov modeling^17^ and clinical experience with pretreatment disease^18^ and fast progressions^19^ due to differences in cycle length. A standard discount rate of 3% was used throughout the analysis.

### Health Costs and Utilities Definitions

We analyzed data from the perspective of the US, Brazilian, Singaporean, and Spanish systems (Table 1; eTables 1-3 in Supplement 1). We obtained country-specific costs regarding diagnosis, drug acquisition, drug administration, monitoring, supportive care, and end-of-life costs as appropriate and available. Diagnosis costs included imaging (eg, positron emission tomography/computed tomography scans) and immunohistochemical staining. Drug acquisition costs were based on country-specific data either widely available on the internet or as a result of local contracts with national hospitals as of August 15, 2023. Drug administration costs incorporated dose timing and intravenous administration per hour. We used median PFS as a median treatment duration for each arm. The cost of postprogression therapies was also assessed according to the number of patients receiving each line of therapy according to biomarker status per the KEYNOTE-189,^5^ FLAURA,^7^ ALEX,^8^ and REVEL^6^ trials. Monitoring costs included imaging (eg, positron emission tomography/computed tomography scans). End-of-life costs were variable by country, including, for example, death costs according to previous analyses in the US case^14^ (Table 1) or average best-case daily costs for inpatient monitoring in the Spanish case^20^ (eTable 3 in Supplement 1).

In addition to drug acquisition and administration costs, we considered the costs of adverse events and supportive care according to published data^15^ (Table 1). Adverse event costs were used from previously established analyses according to the prevalence of toxic effects reported in each drug’s randomized phase 3 trial leading to regulatory approval. Supportive care consisted of surveillance visits for either toxic effects management or long-term surveillance without active therapy administered.

Brazilian reals were adjusted based on 2021 purchasing power parity exchange rates ($R2.53 reals to 1 US dollar [USD]) provided by the World Health Organization and international markets.^21^ Singaporean dollars (S$)and Euros (€) were converted to USD to facilitate global cost standardization, based on applicable exchange rates at the time of analysis: S$1.34 and €0.90 to $1.

The utility of each health state (PFS, POD1, POD2, and death), as well as disutility and cost of each relevant adverse event (eg, diarrhea, fatigue, dyspnea, stomatitis, rash, anemia, and/or vomiting), were obtained from previous analyses.^14^ Specifically, the baseline utility of PFS was listed as 0.79, POD1 and POD2 as 0.76, and death as 0. We assumed identical utilities for POD1 and POD2 due to a deficit of quality-of-life indicators both before and during the immunotherapy era.^22^

### Study End Points

The study’s primary end point was the incremental cost-effectiveness ratio (ICER) expressed as cost per quality-adjusted life-year (QALY) gained through the use of durvalumab compared with placebo maintenance therapy. Secondary end points included the incremental costs of life-years saved, total costs, drug costs, progression-free drug costs, postprogression drug costs, and adverse event costs.

### Clinical Effectiveness and Quality-of-Life Measures

**Figure 1.**
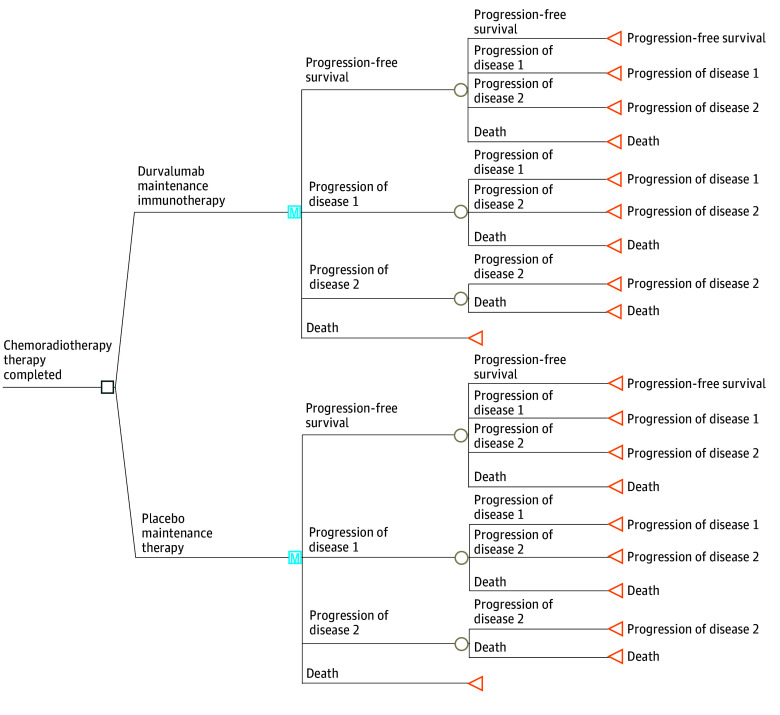
Detailed Markov Model for Treatment Strategies With and Without Maintenance Durvalumab After Consolidative Chemoradiotherapy for Patients With Unresectable Stage III Non–Small Cell Lung Cancer Patients simulated in the model were randomized 1:1 through the durvalumab vs placebo arms and then progressed according to long-term results based on appropriate biomarkers.

In our Markov model, patients were classified into 4 mutually exclusive health states: PFS, POD1, POD2, and death (Figure 1). Individual patient data regarding PFS and OS within the durvalumab and other standard-of-care groups were extracted via previously described techniques, and Kaplan-Meier curves were generated using WebPlotDigitizer (Automeris LLC). For each end point (PFS and OS) and each treatment arm (durvalumab and placebo), event-time distribution was estimated via a composite model^23^ using the nonparametric Kaplan-Meier survival estimator up to last follow-up and the piecewise exponential model for the tail after follow-up.

### Deterministic Sensitivity Analyses

We performed several 1-way deterministic sensitivity analyses to test the robustness of the results and assess strategies that might improve cost-effectiveness. We also conducted probabilistic sensitivity analyses to evaluate the probability of durvalumab being cost-effective on a willingness-to-pay (WTP) threshold of $150 000 per QALY in the US or country-specific thresholds for Brazil ($22 251; 3 times gross domestic product per capita^24^), Singapore ($55 288; S$75 000^25^), and Spain ($107 069; €100,000) based on accepted levels in the literature.

## Results

**Figure 2.**
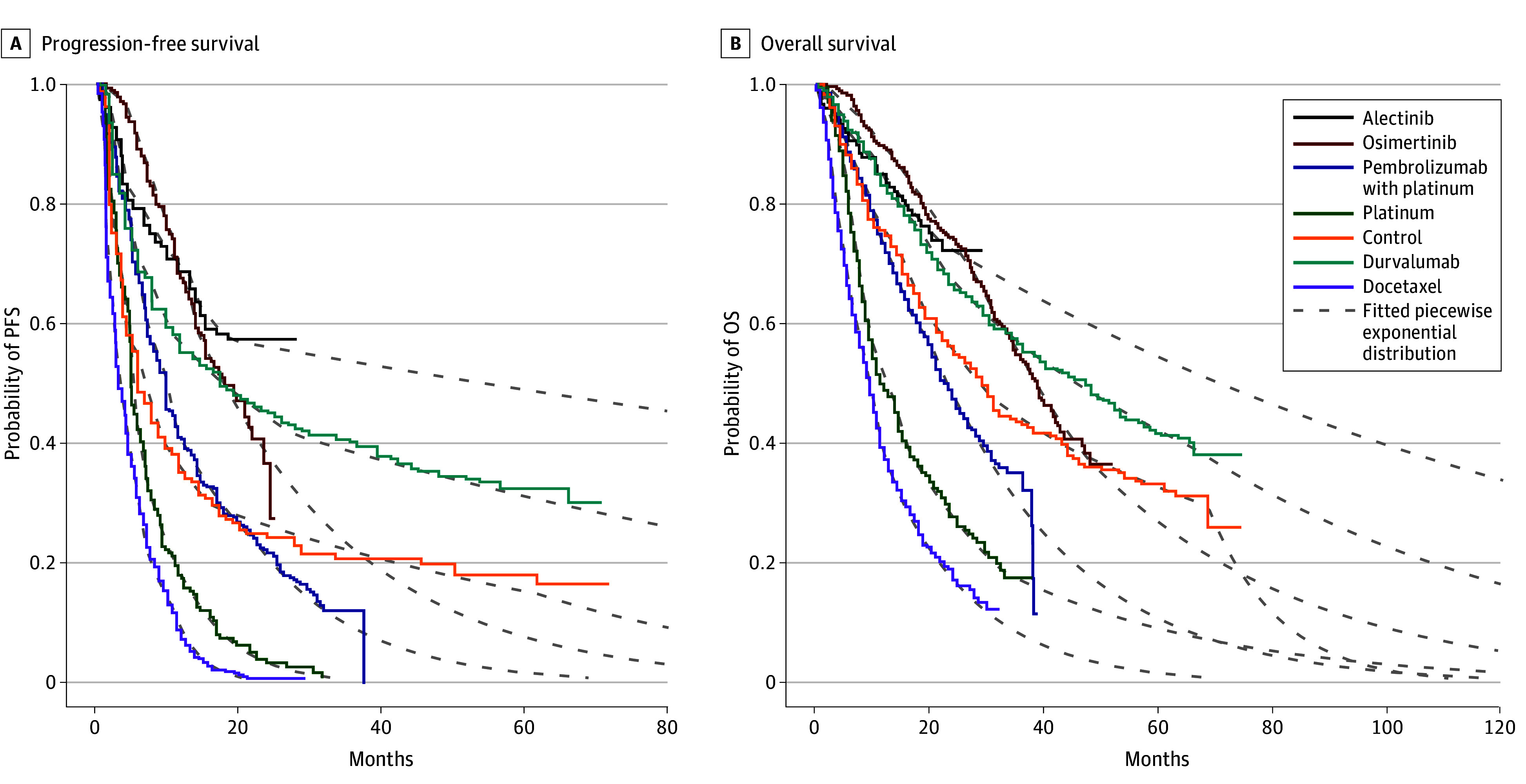
Distributions of Progression-Free Survival (PFS) and Overall Survival (OS) Median PFS (A) and OS (B) estimates, along with fitted piecewise exponential distributions, for each treatment arm used in the Markov model.

For all countries simulated, respective median OS time was 45.0 (95% CI, 42.5-47.5) months and median PFS time was 31.9 (95% CI, 29.0-34.8) months for durvalumab, whereas median OS time was 37.4 (95% CI, 33.7-41.1) months and median PFS time was 19.9 (95% CI, 16.2-23.4) months for placebo (Figure 2). In the US base case, median OS time was 51.1 (95% CI, 48.8-53.4) months and PFS time was 37.9 (95% CI, 35.4-40.5) months for durvalumab vs median OS time of 39.3 (95% CI, 37.5-41.0) months and PFS time of 19.9 (95% CI, 18.2-21.5) months for placebo (Table 2). The incremental number of QALYs gained with durvalumab compared with placebo in the US base case as defined by the PACIFIC trial was 0.50 (95% CI, 0.45-0.55) (Table 2).

**Table 2.** US, Brazil, Singapore, and Spain Base-Case Analyses of Costs for Durvalumab vs Placebo With Incremental Changes^a^

Country-specific base case	Durvalumab	Placebo	Incremental
**US**			
Total costs, $	154 601 (151 441 to 157 760)	40 207 (38 538 to 41 876)	114 394 (111 401 to 117 386)
Drug costs, $	150 914 (147 480 to 154 048)	35 601 (34 058 to 37 143)	115 313 (112 349 to 118 276)
Progression-free drug costs, $	123 254 (120 186 to 126 320)	NA	123 254 (120 186 to 126 320)
Postprogression drug costs, $	27 661 (26 057 to 29 264)	35 601 (34 058 to 37 143)	−7940 (−9000 to −6880)
Adverse event costs, $	3687 (3320 to 4052)	4606 (4203 to 5008)	−919 (−1434 to −404)
PFS, mo	37.9 (35.4 to 40.5)	19.9 (18.2 to 21.5)	18.0 (16.9 to 19.3)
OS, mo	51.1 (48.8 to 53.4)	39.3 (37.5 to 41.0)	11.8 (10.6 to 13.0)
QALY, y	2.72 (2.61 to 2.83)	2.22 (2.13 to 2.31)	0.50 (0.45 to 0.55)
Incremental cost per life-year, $	NA	NA	116 332 (105 594 to 129 502)
Durvalumab ICER per QALY, $	228 788 (207 989 to 254 208)	NA	NA
**Brazil**			
Total costs, $	112 678 (109 353 to 116 001)	44 928 (41 578 to 48 277)	67 750 (65 329 to 70 168)
Drug costs, $	109 432 (106 124 to 112 739)	40 631 (37 290 to 43 971)	68 801 (66 450 to 71 151)
Progression-free drug costs, $	76 492 (74 622 to 78 360)	NA	76 492 (74 622 to 78 360)
Postprogression drug costs, $	32 940 (29 848 to 36 031)	40 631 (37 290 to 43 971)	−7691 (−9564 to −5817)
Adverse event costs, $	3246 (2894 to 3597)	4297 (3889 to 4704)	−1051 (−1556 to −546)
PFS, mo	37.9 (35.4 to 40.5)	19.9 (18.2 to 21.5)	18.0 (16.9 to 19.3)
OS, mo	52.7 (50.3 to 55.0)	41.4 (39.6 to 43.2)	11.3 (10.1 to 12.5)
QALY, y	2.80 (2.69 to 2.91)	2.32 (2.23 to 2.42)	0.48 (0.43 to 0.53)
Incremental cost per life-year, $	NA	NA	71 947 (65 040 to 80 495)
Durvalumab ICER per QALY, $	141 146 (127 830 to 157 558)	NA	NA
**Singapore**			
Total costs, $	120 566 (116 823 to 124 308)	49 974 (46 175 to 53 771)	70 592 (68 040 to 73 143)
Drug costs, $	117 801 (114 061 to 121 540)	46 202 (42 397 to 50 005)	71 599 (69 094 to 74 104)
Progression-free drug costs, $	82 296 (80 285 to 84 307)	NA	82 296 (80 285 to 84 307)
Postprogression drug costs, $	35 505 (31 989 to 39 020)	46 202 (42 397 to 50 005)	−10 697 (−12 648 to −8744)
Adverse event costs, $	2764 (2442 to 3086)	3772 (3397 to 4146)	−1008 (−1491 to −523)
PFS, mo	37.9 (35.4 to 40.5)	19.9 (18.2 to 21.5)	18.0 (16.9 to 19.3)
OS, mo	53.1 (50.8 to 55.4)	42.0 (40.2 to 43.8)	11.1 (9.2 to 12.2)
QALY, y	2.82 (2.72 to 2.93)	2.36 (2.27 to 2.45)	0.46 (0.42 to 0.52)
Incremental cost per life-year, S	NA	NA	76 315 (69 434 to 92 076)
Durvalumab ICER per QALY, $	153 460.9 (135 753 to 168 076)	NA	NA
**Spain**			
Total costs, $	109 777 (105 970 to 113 583)	43 425 (39 515 to 47 335)	66 352 (63 752 to 68 951)
Drug costs, $	106 108 (102 362 to 109 853)	39 026 (35 187 to 42 863)	67 082 (64 557 to 69 607)
Progression-free drug costs, $	76 621 (74 748 to 78 492)	NA	76 621 (74 748 to 78 492)
Postprogression drug costs, $	29 487 (26 076 to 32 898)	39 026 (35 187 to 42 863)	−9539 (−11 578 to −7497)
Adverse event costs, $	3669 (3299 to 4038)	4400 (4013 to 4786)	−731 (−1240 to −221)
PFS, mo	37.9 (35.4 to 40.5)	19.9 (18.2 to 21.5)	18.0 (16.9 to 19.3)
OS, mo	50.8 (48.5 to 53.1)	38.4 (36.7 to 40.1)	12.4 (11.2 to 13.5)
QALY, y	2.71 (2.60 to 2.82)	2.18 (2.09 to 2.27)	0.53 (0.48 to 0.58)
Incremental cost per life-year, S	NA	NA	64 212 (58 979 to 71 091)
Durvalumab ICER per QALY, $	125 193 (114 400 to 138 233)	NA	NA

Abbreviations: ICER, incremental cost-effectiveness ratio; NA, not applicable; OS, overall survival; PFS, progression-free survival; QALY, quality-adjusted life-year.

^a^ The mean value for each variable was reported based on 1000 simulated individual patient data with a specific random seed.

All country-specific base cases demonstrated cost-prohibitive (ie, ICER greater than country-specific, established WTP threshold) durvalumab pricing. In the US, the durvalumab ICER per QALY was $228 788 (95% CI, $207 989-$254 208) compared with placebo in the base case. The incremental cost per 1 life-year saved was $116 332 (95% CI, $105 594-$129 502). In Brazil, the durvalumab ICER per QALY was $141 146 (95% CI, $127 830-$157 558) compared with placebo in the base case. The incremental cost per 1 life-year saved was $71 947 (95% CI, $65 040-$80 495). In Singapore, the ICER per QALY was $153 461 (95% CI, $135 753-$168 076) compared with placebo in the base case. The incremental cost per 1 life-year saved was $76 315 (95% CI, $69 434-$92 076). In Spain, the ICER per QALY was $125 193 (95% CI, $114 400-$138 233) compared with placebo. The incremental cost per 1 life-year saved was $64 212 (95% CI, $58 979-$71 091).

**Figure 3.**
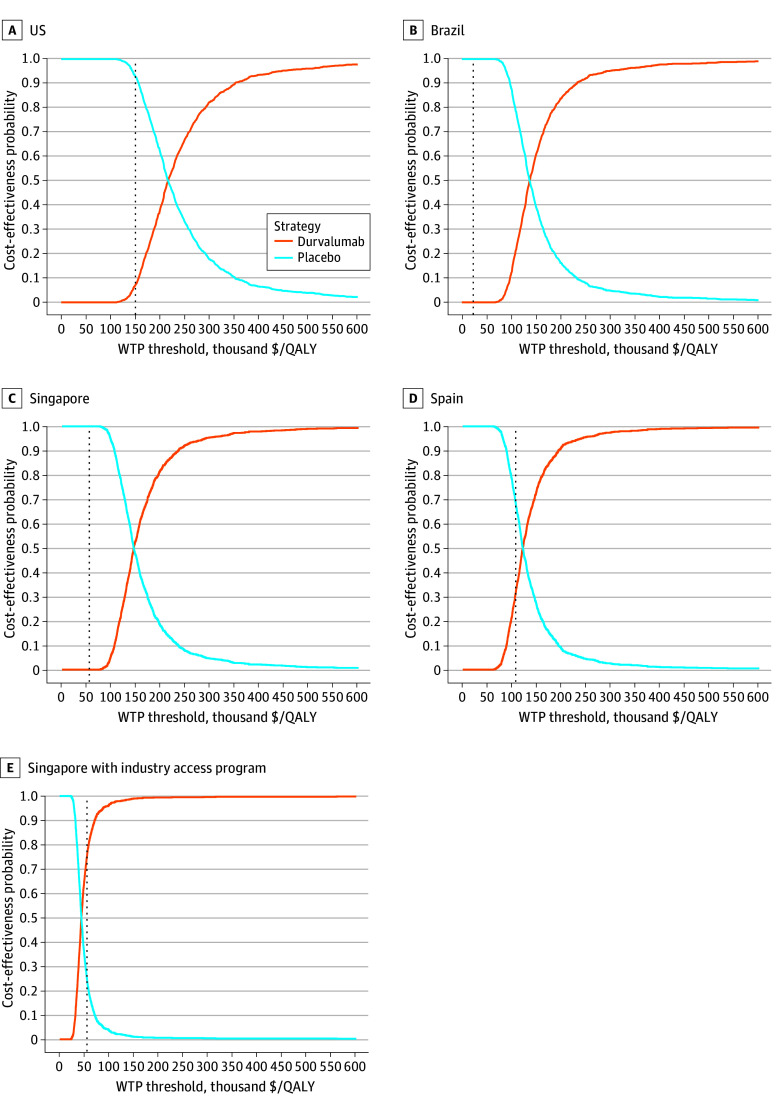
Cost-Effectiveness Acceptability Curves According to the Base Cases Acceptability curves shown for the US (A), Brazil (B), Singapore (C), and Spain (D), as well as in an industry access program (E). The dotted line represents the willingness-to-pay (WTP) threshold according to each country’s established baseline. Results of the probabilistic sensitivity analysis are based on 1000 Monte Carlo simulations. The acceptability curves show the proportion of incremental cost-effectiveness ratios that are acceptable according to each WTP threshold. QALY indicates quality-adjusted life-year.

Tornado diagrams of 1-way deterministic sensitivity analyses showed that the strongest factor on incremental QALY was the utility of PFS of durvalumab in all countries (eTable 4, eFigure in Supplement 1). The cost of durvalumab was only the fourth strongest factor in our deterministic sensitivity analyses. In the probabilistic sensitivity analyses considering current costs and WTP thresholds, we found that durvalumab is unlikely to be cost-effective in all 4 countries (7.2% in the US, 0% in Brazil, 0% in Singapore, and 31% probability in Spain) (Figure 3).

A secondary analysis using discounted prices via an industry-sponsored access program in Singapore yielded cost-effective results. In this access program, the durvalumab, 500-mg, vial is offered at a buy-1-get-2-free monthly rate, while the 120-mg vial is offered at a buy-2-get-remainder-free monthly adjustment. At a S$1000/vial cost, the average monthly $6.38/mg cost is therefore discounted 33.7% to $4.23/mg (based on a 70-kg patient). In this case, durvalumab has a 74.9% cost-effective probability (Figure 2). The incremental cost per 1 life-year saved was $23 290 (95% CI, $21 189-$28 855). Durvalumab price adjustments to the PACIFIC data improved cost-effectiveness in Singapore, with an ICER of $45 164.

## Discussion

The initiation of immunotherapy has prolonged 5-year life expectancy for patients with NSCLC. In our analysis, we chose to examine the cost-effectiveness of durvalumab as maintenance therapy after combined chemoradiotherapy in unresectable stage III NSCLC in high-income (US, Singapore, and Spain) and middle-income (Brazil) countries within 4 geographic regions (North America, South America, Southeast Asia, and Europe). To our knowledge, this analysis represents the latest and most comprehensive evaluation of durvalumab using 5-year outcomes data in multiple clinical practice country frameworks.

### Global Barriers to Use of Durvalumab

While cost has been cited as one of the major barriers of the global use of durvalumab, others merit discussion. First, health systems approvals do not uniformly approve durvalumab in the treatment of locally advanced, unresectable NSCLC. For example, the European Medicines Agency only permits durvalumab for patients whose tumor proportions scores are 1% or more based on post hoc, unplanned data released after the PACIFIC trial was published. A retrospective analysis of the Spanish Thoracic Tumor Registry in the 2010s reported that chemoradiotherapy remains the most common treatment modality for patients with stage III NSCLC in greater than one-third of patients, amounting to more than 6000 patients for whom durvalumab may be indicated.^26^ The number of cases that do not qualify for durvalumab based on PD-L1 tumor proportion score remains unknown.

The next obstacle to the broad uptake of durvalumab globally relates to patient access. The RELIANCE study retrospectively analyzed patients with stage III NSCLC in Brazil and found that only 9 of 403 patients (2.2%) with locally advanced disease had access to durvalumab as treatment in clinical trials between 2015 and 2019, as durvalumab was approved in Brazil in 2020.^27^ Nevertheless, the treatment remains unreimbursed in the public health system, through which more than two-thirds of the Brazilian population obtains health care—although cost-effectiveness analyses remain under consideration by the public Sistema Único de Saúde system.^28^

In addition, patient eligibility remains a key barrier globally. Despite worldwide consensus that PD-L1 tumor proportion score biomarker status is a necessary treatment consideration, not all patients receive such testing. For example, patients diagnosed through pleural fluid cytologic samples rather than tissue biopsy might yield insufficient samples to perform testing.^29^ Greater awareness and knowledge of these and other barriers might improve the use of durvalumab. Previous as well as ongoing efforts, such as the International Association of the Study of Lung Cancer’s Global Survey on Molecular Testing in Lung Cancer^30^ and Global Survey on Biomarker Testing in Lung Cancer,^31^ aim to eradicate these challenges.

### Implications of Costs of Immunotherapy

The implications of immunotherapy pricing affect all levels of the health system, from those of the patient and clinician to that of national or international budgets. Whenever countries dedicate portions of finite budgets to cover immunotherapy, both health and opportunity costs must be delicately balanced. The UK’s National Institute for Health and Care Excellence currently limits approval to new therapies based on a cost-benefit threshold of £20 000 to £30 000 (approximately $25 000-$39 000) per QALY.^32^ Atezolizumab, an immunotherapy similar to durvalumab that targets PD-L1, is accordingly approved in the metastatic NSCLC setting^33^ in both intravenous and subcutaneous formulations,^34^ although the agent does not retain such approval in small-cell lung cancer due to a higher estimated ICER. Finland, another high-income European country, has also declined to approve durvalumab in extensive-stage small-cell lung cancer due to low efficacy and high costs.^35^ These budgetary decisions are often personalized to the country and its population. Furthermore, there is a call for more equitable benefit-cost analyses that also factor social determinants of health^36^ when producing these decisions to decrease the no-value or low-value^37^ use of immunotherapy.

### Our Model Within the Context of Previous Analyses

Previous analyses using WTP thresholds found durvalumab to be cost-effective with ICER per QALY in the US ($100 00),^14^ Swiss (CHF100 000 [ ~ $111 330]),^38^ and Chinese ($150 000)^39^ systems. Our analysis, bolstered by long-term outcomes data, suggests that durvalumab pricing remains cost-prohibitive globally. One explanation for our findings is that the population of countries not studied as extensively in the PACIFIC trial may have far lower median percentages of PD-L1–positive tumors and/or higher percentages of *EGFR*-mutant NSCLC, such as in Brazil,^40^ or *ALK*-rearranged NSCLC, such as in Singapore.^41^

Differences in model inputs according to country may also explain part of the variance that we observed between country cases. For example, biomarker positivity varied greatly among populations, with absolute lows of 6% for *EGFR* and 1.12% for *ALK* in the US to absolute highs of 43.4% for *EGFR* in Singapore and 11% for *ALK* in Spain. Our sensitivity analyses otherwise controlled for model inputs related to statistical uncertainty regarding utilities or costs. In line with previous analyses,^38,39^ our deterministic sensitivity analyses found that the cost of durvalumab itself was not as influential on the likelihood of cost-effectiveness compared with the PFS utility of durvalumab treatment.

### Ramifications on Pricing Strategy

The primary conclusion of our analysis is the increased cost-effectiveness of an industry-sponsored access program to convert cost-prohibitive therapies into cost-effective treatments. Care should be used by industry and other sellers of oncology drugs to maintain the proposed increased access after market share is obtained, especially in public hospitals in low- and middle-income countries.^42^ On the regulatory side, expedited approvals in high-income countries, such as the US, can lead to more rapid approvals and access in low- and middle-income countries, such as Brazil, although it is unclear whether this strategy results in achieving cost-effective pricing.^43^ In addition, value-based pricing is an alternative model used by payers that industry might adapt to inform global pricing strategies that minimize financial toxicity and increase global access to oncology drugs.^44^

### Strengths and Limitations

Strengths of our model include its timeframe, use of multiple lines of therapy, and biomarker-guided treatment paradigm. A notable limitation of our analysis relates to utilities estimates within the immunotherapy era. As stated herein, there is no consensus related to updated utilities that adequately reflect treatment using immune checkpoint inhibitors. For example, a previous meta-analysis identified only 1 disutility of −0.043 for patients receiving intravenous chemotherapy in the second-line setting of metastatic NSCLC; this estimate is based on an industry submission to the National Institute for Health and Care Excellence in 2006 while evaluating erlotinib.^45^ Therefore, contemporary cost-effectiveness analyses such as ours might standardize utilities to decrease bias. Our assumed POD1 and POD2 utility of 0.76 aligns well with other disparate estimates in the field, with a meta-analysis citing utility ranges of 0.66 to 0.84 in stage IV NSCLC.^46^ Alternative methods that might minimize bias introduction within NSCLC specifically include utilities analyzed by time to death, although this method has not yet gained traction.^47^

Another major limitation of our model includes the reliance on utilities and QALYs in oncologic cost-effectiveness analyses. While these economic estimates aim to standardize health-state preferences, they inherently vary based on the population of interest. For example, WTP/QALY ratios were found to vary greatly related to patients’ social determinants of health characteristics, such as family income, area income, educational level, and number of coinhabitants when factored into Spanish QALY surveys.^48^ Moreover, Spanish oncologists treating patients with cancer do not uniformly agree with the traditional €100 000 ICER cited in cost-effectiveness analyses, with 31.2% finding costs above that threshold as acceptable.^49^

Estimates of QALYs also vary by clinical trial design and preference. For example, patient-reported outcomes of the PACIFIC trial were estimated using the European Organization for Research and Treatment of Cancer Quality of Life Questionnaire–Core 30 and Quality of Life Questionnaire–Lung Cancer 13 methods,^50^ whereas patient-reported outcomes of the REVEL trial were not initially reported but later published in a post hoc analysis stratified by front-line therapy using the Lung Cancer Symptom Scale questionnaire.^51^ Such variations will hopefully decrease over time as the field reaches consensus on optimal patient-reported outcomes data collection. In addition, our model lacks the inclusion of low-income, African, or Oceanic countries.

## Conclusions

In this economic evaluation, durvalumab was not cost-effective in our model in any of the 4 countries studied despite being highly efficacious in the maintenance setting after consolidative chemoradiotherapy for unresectable stage III NSCLC. In Singapore, the institution of an industry-sponsored access program greatly decreased drug costs, thereby converting durvalumab into a cost-effective therapy.

## Notes

Supplement 1. **eTable 1.** Inputs and References Corresponding With the Markov Model’s Patient Characteristics and Costs Used in the Markov Model: Brazil Base Case
eReferences 1.
**eTable 2.** Inputs and References Corresponding With the Markov Model’s Patient Characteristics and Costs: Singapore Base Case
eReferences 2.
**eTable 3.** Inputs and References Corresponding With the Markov Model’s Patient Characteristics and Costs: Spain Base Case
eReferences 3.
**eTable 4.** Sensitivity Analysis Parameters That Were Used to Ensure the Reliability of Our Markov Model’s Findings **eFigure.** Tornado Diagrams of One-Way Sensitivity Analysis

Supplement 2.
Data Sharing Statement
