# Supplementary material for: International Cost-Effectiveness Analysis of Durvalumab in Stage III Non–Small Cell Lung Cancer
Source: JAMA Netw Open. 2024 May 30;7(5):e2413938. doi: 10.1001/jamanetworkopen.2024.13938 (PMC11140532; doi:10.1001/jamanetworkopen.2024.13938)
Supplement: Supplement 1. — eTable 1. Inputs and References Corresponding With the Markov Model’s Patient Characteristics and Costs Used in the Markov Model: Brazil Base Case eReferences 1. eTable 2. Inputs and References Corresponding With the Markov Model’s Patient Characteristics and Costs: Singapore Base Case eReferences 2. eTable 3. Inputs and References Corresponding With the Markov Model’s Patient Characteristics and Costs: Spain Base Case eReferences 3. eTable 4. Sensitivity Analysis Parameters That Were Used to Ensure the Reliability of Our Markov Model’s Findings eFigure. Tornado Diagrams of One-Way Sensitivity Analysis [file jamanetwopen-e2413938-s001.pdf]

Supplementary Online Content

Kareff SA, Han S, Haaland B, et al. International cost-effectiveness analysis of durvalumab in stage III non–small cell lung cancer. *JAMA Netw Open*. 2024;7(5):e2413938. doi:10.1001/jamanetworkopen.2024.13938

eTable 1. Inputs and References Corresponding With the Markov Model's Patient Characteristics and Costs Used in the Markov Model: Brazil Base Case

eReferences 1

eTable 2. Inputs and References Corresponding With the Markov Model's Patient Characteristics and Costs: Singapore Base Case

eReferences 2

eTable 3. Inputs and References Corresponding With the Markov Model's Patient Characteristics and Costs: Spain Base Case

eReferences 3

eTable 4. Sensitivity Analysis Parameters That Were Used to Ensure the Reliability of Our Markov Model's Findings

eFigure. Tornado Diagrams of One-Way Sensitivity Analysis

This supplementary material has been provided by the authors to give readers additional information about their work.

eTable 1. Inputs and References Corresponding With the Markov Model's Patient Characteristics and Costs: Brazil Base Case

Variable	Input	Reference
Patient Characteristics		
Squamous histology (%)	44.4	1
Non-squamous histology (%)	55.6	1
PD-L1 TPS ≥ 1% (%)	42.5	2
PD-L1 TPS < 1% (%)	20.8	2
PD-L1 unknown (%)	36.7	2
EGFR mutant (%)	16.5	1
ALK rearranged (%)	2.4	1
Costs (converted to USD)		
Durvalumab (1mg)	5.93	3
Pembrolizumab (1mg)	35.58	3
Pemetrexed (1mg)	2.33	3
Carboplatin (50mg)	0.95	3
Docetaxel (1mg)	7.73	3
Nab-paclitaxel (1mg)	2.22	3
Osimertinib (80mg; monthly)	7,205.70	3
Alectinib (600mg; monthly)	5,685.60	3
Imaging/surveillance (i.e.., PET/CT)	552.93	4
Immunohistochemical testing (IHC 1st stain)	108.26	4
Radiotherapy (i.e. 60 Gy, IMRT)	7,496.77	4
Monthly best supportive care	17.73	4
End-of-life costs	1,034	5
Willingness-to-Pay Threshold (WTP)	22,251 (3x G.D.P. per capita)	6

Legend: This table lists the various inputs and references corresponding with the Markov model's patient characteristics and costs used in the Markov model. The values are all according to the Brazil base case (converted to and listed in USD).

© 2024 Kareff SA et al. *JAMA Network Open.*

eReferences 1

1. Kelner M, Carvalho da Silva B, Montella T, et al. Discrepancies Between the Cost of Advanced Lung Cancer Treatment and How Much Is Reimbursed by the Brazilian Public Healthcare System. *Value Health Reg Issues*. 2023;33:1-6. doi:10.1016/j.vhri.2022.08.004

2. Spigel DR. Five-Year Survival Outcomes From the PACIFIC Trial: Durvalumab After Chemoradiotherapy in Stage III Non-Small-Cell Lung Cancer. *J Clin Oncol*. 2022;20(40):1301-1311. doi:10.1200/JCO.21.01308. Epub 2022 Feb 2

3. Agência Nacional de Vigilância Sanitária - Anvisa. Câmara de Regulação do Mercado de Medicamentos (CMED). Published online July 2022. Accessed July 5, 2023. https://www.gov.br/anvisa/pt-br/assuntos/medicamentos/cmed

4. Associação Médica Brasileira. Classificação Brasileira Hierarquizada de Procedimentos Médicos. Published online July 2022. Accessed July 5, 2023. https://amb.org.br/cbhpm/

5. Aguiar PN. Cost-effectiveness of Osimertinib in the First-Line Treatment of Patients With EGFR-Mutated Advanced Non–Small Cell Lung Cancer. *JAMA Oncol*. 2018;4(8):1080-1084. doi:doi:10.1001/jamaoncol.2018.1395

6. Soarez PCD, Novaes HMD. Limiares de custo-efetividade e o Sistema Único de Saúde. *Cad Saúde Pública*. 2017;33(4). doi:10.1590/0102-311x00040717

© 2024 Kareff SA et al. *JAMA Network Open.*

eTable 2. Inputs and References Corresponding With the Markov Model's Patient Characteristics and Costs: Singapore Base Case

**Variable**	**Input**	**Reference**
Patient Characteristics		
Squamous histology (%)	29.8	[1]
Non-squamous histology (%)	70.2	[1]
PD-L1 TPS ≥1% (%)	59.4	[2]
PD-L1 TPS <1% or unknown (%)	40.6	[2]
EGFR mutant (%)	43.4	[2]
ALK rearranged (%)	2	[3]
Costs (converted to USD)		
Durvalumab (1mg)	6.38	[4]
Durvalumab (patient access pricing, based on 70kg patient)	4.23	[5]
Pembrolizumab (1mg)	36.99	[4]
Pemetrexed (1mg)	0.12	[4]
Carboplatin (50mg)	0.06	[4]
Docetaxel (1mg)	0.72	[4]
Paclitaxel (1mg)	0.10	[4]
Osimertinib (80mg; monthly)	6,039.6	[4]
Osimertinib (patient access pricing)	3,028.5	[5]
Alectinib (600mg; monthly)	5,496	[4]
Monitoring cost	64.18	[4]
Drug administration per hour (i.e.., chemo infusion 1 hour)	53.79	[4]
Monthly best supportive care	64.18	[6]
End-of-life costs	3,383	[6]
Willingness to Pay (WTP) Threshold	55,288 (S$75,000)	[7]

Legend: This table lists the various inputs and references corresponding with the Markov model's patient characteristics and costs. The values are all according to the Singapore base case (converted to and listed in USD). **Note:** Singapore costs are largely estimated on National University Hospital of Singapore internal pricing after governmental subsidies (Reference 4) and a Patient Access Program sponsored by Astra Zeneca in Singapore (Reference 5).

© 2024 Kareff SA et al. *JAMA Network Open.*

eReferences 2

1. Huang Y, Zhao JJ, Soon YY, et al. Real-world experience of consolidation durvalumab after concurrent chemoradiotherapy in stage III non-small cell lung cancer. *Thorac Cancer*. 2022;13(22):3152-3161. doi:10.1111/1759-7714.14667

2. Soo R, Byoung-Chul C, Prabhash K, et al. Treatment Patterns and Outcomes in Stage III Non-small Cell Lung Cancer (NSCLC): Real-world Experience in Singapore From the KINDLE Study. Poster presented at: ESMO 2020; November 2020; Virtual. Accessed August 15, 2023. https://doi.org/10.1016/j.annonc.2020.10.365

3. Loh C, Koh W, Ang J, Lee W, Chew W, Koh M. Characteristics of Singapore lung cancer patients who miss out on lung cancer screening recommendations. *Singapore Med J*. Published online April 3, 2022. doi:10.11622/smedj.2022039

4. National University Hospital of Singapore Pricing. Published online August 15, 2023.

5. Astra Zeneca Patient Access Program in Singapore. Published online August 15, 2023.

6. Doble B, Wong WHM, Finkelstein E, COMPASS study team. End-of-life cost trajectories and the trade-off between treatment costs and life-extension: Findings from the Cost and Medical Care of Patients with Advanced Serious Illness (COMPASS) cohort study. *Palliat Med*. 2021;35(5):893-903. doi:10.1177/0269216321999576

7. Viswambaram A, Wee YR, Lim S. PMU20 Is There an Implicit Willingness-to-Pay Threshold in Singapore? *Value Health Reg Issues*. 2020;22:S72. doi:10.1016/j.vhri.2020.07.378

© 2024 Kareff SA et al. *JAMA Network Open.*

eTable 3. Inputs and References Corresponding With the Markov Model's Patient Characteristics and Costs: Spain Base Case

Variable	Input	Reference
Patient Characteristics		
Squamous histology (%)	30.3	[1]
Non-squamous histology (%)	69.7	[1]
PD-L1 TPS ≥1% (%)	62.3	[2]
PD-L1 TPS <1% or unknown (%)	37.7	[2]
EGFR mutant (%)	18	[1]
ALK rearranged (%)	11	[1]
Costs (converted to USD)		
Durvalumab (1mg)	5.94	[3]
Pembrolizumab (1mg)	39.27	[3]
Pemetrexed (1mg)	0.11	[3]
Carboplatin (50mg)	0.17	[3]
Docetaxel (1mg)	2.42	[3]
Nab-paclitaxel (1mg)	2.64	[3]
Osimertinib (80mg; monthly)	6,673.70	[3]
Alectinib (600mg; monthly)	6,175.77	[3]
Imaging/surveillance (i.e.., PET/CT)	209	[4]
Immunohistochemical testing (IHC 1st stain)	66	[4]
Radiotherapy (i.e. 60 Gy, IMRT)	5775	[4]
Monthly best supportive care	104.5	[4]
End-of-life costs (average)	386.4	[5]
Willingness-to-pay Threshold	107,069 (€100,000)	NA

Legend: This table lists the various inputs and references corresponding with the Markov model's patient characteristics and costs. The values are all according to the Spain base case (converted to and listed in USD). **Note**: Spain costs are largely estimated on the Hospital Universitario Fundación Jiménez Díaz' internal pricing after governmental bargaining (Reference 4).

© 2024 Kareff SA et al. *JAMA Network Open.*

eReference 3

1. Remon J, Reguart N, García-Campelo R, et al. Lung Cancer in Spain. *J Thorac Oncol Off Publ Int Assoc Study Lung Cancer*. 2021;16(2):197-204. doi:10.1016/j.jtho.2020.09.026

2. Saez de Gordoa K, Lopez I, Marginet M, et al. PD-L1 Expression in Non-Small Cell Lung Cancer: Data from a Referral Center in Spain. *Diagn Basel Switz*. 2021;11(8):1452. doi:10.3390/diagnostics11081452

3. Información sobre los productos incluidos en la prestación farmacéutica del SNS (dispensables a través de oficinas de farmacia). Published online June 2023. Accessed June 24, 2023. https://www.sanidad.gob.es/profesionales/nomenclator.do

4. I. COMUNIDAD DE MADRID A) Disposiciones Generales Consejería de Sanidad. Published online August 21, 2017. Accessed June 23, 2023. https://www.bocm.es/boletin/CM_Orden_BOCM/2017/08/21/BOCM-20170821-1.PDF

5. Herrera Abian M. End of Life Cost Savings in the Palliative Care Unit Compared to Other Services. *J Pain Symptom Manage*. 2022;64(5):495-503. doi:10.1016/j.jpainsymman.2022.06.016

© 2024 Kareff SA et al. *JAMA Network Open.*

eTable 4. Sensitivity Analysis Parameters That Were Used to Ensure the Reliability of Our Markov Model's Findings

Variable	Baseline	Range	Distribution
US Drug cost (1mg), $			
Durvalumab	7.71	(6.168, 9.252)	Gamma (96.04, 12.5)
Pembrolizumab	53.7	(42.96, 64.44)	Gamma (96.04, 1.8)
Pemetrexed	7.64	(6.112, 9.168)	Gamma (96.04, 12.5)
Nab-paclitaxel	14.87	(11.896, 17.844)	Gamma (96.04, 6.5)
Brazil Drug cost (1mg), $			
Durvalumab	5.93	(4.744, 7.116)	Gamma (96.04, 16)
Pembrolizumab	35.58	(28.464, 42.696)	Gamma (96.04, 2.7)
Pemetrexed	2.33	(1.864, 2.796)	Gamma (96.04, 41)
Nab-paclitaxel	2.22	(1.776, 2.664)	Gamma (96.04, 43)
Singapore Drug cost (1mg), $			
Durvalumab (main analysis)	6.38	(5.104, 7.656)	Gamma (96.04, 15)
Durvalumab (sub analysis)	2.115	(1.692, 2.538)	Gamma (96.04, 45)
Pembrolizumab	36.99	(29.592, 44.388)	Gamma (96.04, 2.6)
Pemetrexed	0.12	(0.096, 0.144)	Gamma (96.04, 800)
Paclitaxel	0.10	(0.08, 0.12)	Gamma (96.04, 950)
Spain Drug cost (1mg), $			
Durvalumab	5.94	(4.752, 7.128)	Gamma (96.04, 16)
Pembrolizumab	39.226	(31.381, 47.071)	Gamma (96.04, 2.5)
Pemetrexed	0.110	(0.088, 0.132)	Gamma (96.04, 900)
Nab-paclitaxel	2.64	(2.112, 3.168)	Gamma (96.04, 36)
Utility			
PFS durvalumab	0.784	(0.627, 0.941)	Beta (50, 13.3)
PFS placebo	0.815	(0.652, 0.978)	Beta (50, 11.5)
POD1	0.760	(0.608, 0.912)	Beta (50, 16)
POD2	0.760	(0.608, 0.912)	Beta (50, 16)
Discount rate, %	3	5 (fixed)	--
Discount rate, %	3	10 (fixed)	--

Legend: This tale lists the sensitivity analysis parameters that were used to ensure the reliability of our Markov model's findings. **Note:** The range for each variable is within a variance of 20% from its baseline value.

© 2024 Kareff SA et al. *JAMA Network Open.*

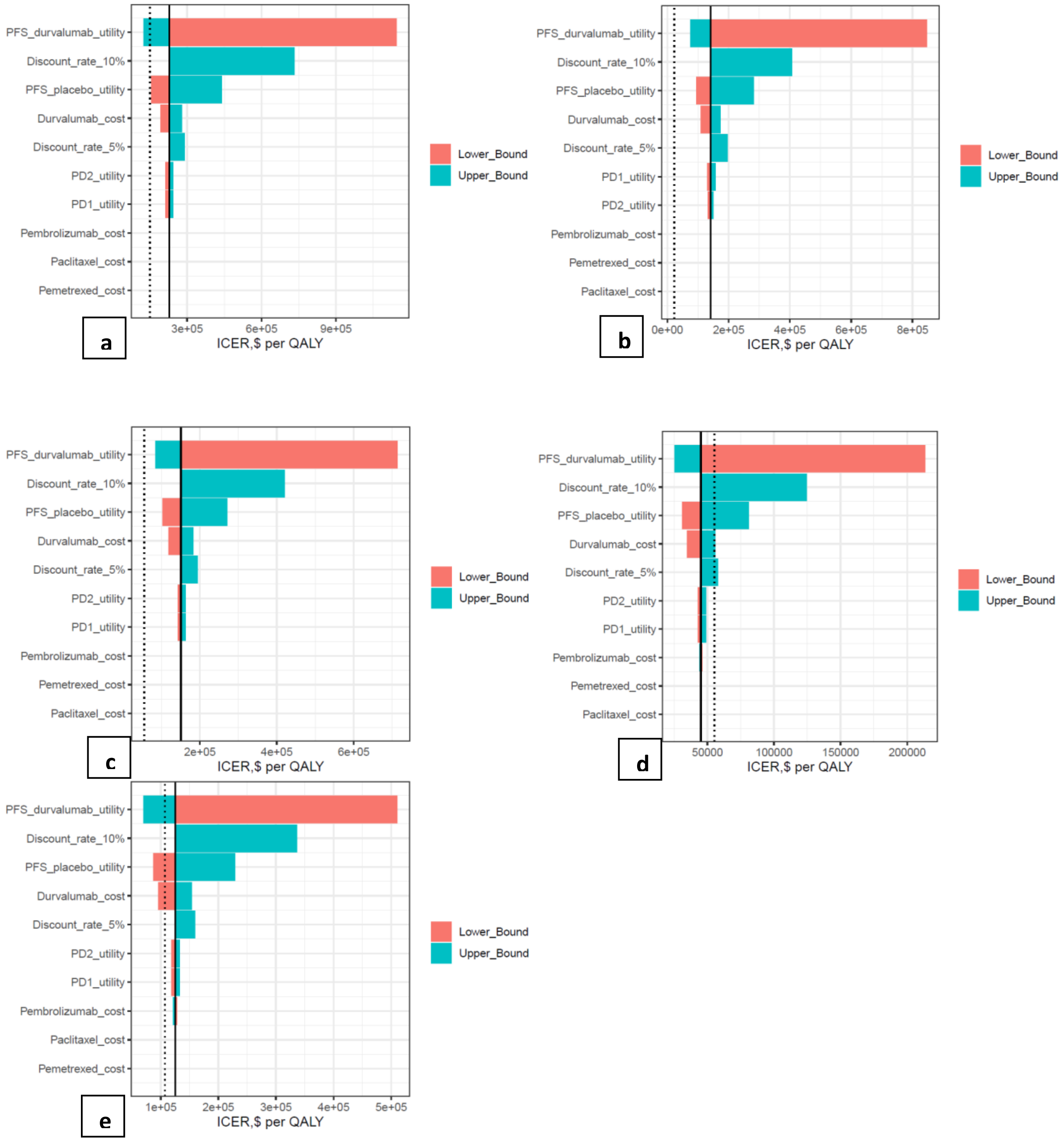

eFigure. Tornado diagrams of one-way sensitivity analysis in the US (**a**), Brazil, (**b**), Singapore (**c**), Singapore with patient access program pricing (**d**), and Spain (**e**).

Legend: The solid line in each diagram represents the country-specific ICER per QALY, and the dotted line represents the country-specific willingness-to-pay threshold per QALY. PFS: progression-free survival; PD1: first progression of disease; PD2: second progression of disease.

© 2024 Kareff SA et al. *JAMA Network Open.*
