# Supplementary material for: International Cost-Effectiveness Analysis of Durvalumab in Stage III Non–Small Cell Lung Cancer
Source: JAMA Netw Open. 2024 May 30;7(5):e2413938. doi: 10.1001/jamanetworkopen.2024.13938 (PMC11140532; doi:10.1001/jamanetworkopen.2024.13938)
Supplement: Supplement 2. — Data Sharing Statement [file jamanetwopen-e2413938-s002.pdf]

Data Sharing Statement

Kareff. International Cost-Effectiveness Analysis of Durvalumab in Stage III Non–Small Cell Lung Cancer. *JAMA Netw Open*. Published May 30, 2024.
doi:10.1001/jamanetworkopen.2024.13938

Data
Data available: Yes
Data types: Data (not involving human participants), Data dictionary
How to access data: Can upload to GitHub if requested by reviewers. Otherwise, primary biostatistician sxh3040@med.miami.edu has access to all data with requests to be fielded by PI, Dr. Lopes.
When available: With publication

Supporting Documents
Document types: Statistical/analytic code
How to access documents: Can upload to GitHub if requested by reviewers. Otherwise, primary biostatistician sxh3040@med.miami.edu has access to all data with requests to be fielded by PI, Dr. Lopes.
When available: With publication

Additional Information
Who can access the data: Anyone requesting the data
Types of analyses: For any purpose
Mechanisms of data availability: Without investigator support
